# Biomolecule-Producing Probiotic Bacterium *Lactococcus lactis* in Free or Nanoencapsulated Form for Endometritis Treatment and Fertility Improvement in Buffaloes

**DOI:** 10.3390/jfb15060138

**Published:** 2024-05-21

**Authors:** Nesrein M. Hashem, Walaa M. Essawi, Azza S. El-Demerdash, Ali Ali El-Raghi

**Affiliations:** 1 Department of Animal and Fish Production, Faculty of Agriculture, Alexandria University, Alexandria 21545, Egypt; 2 Departamento de Produccion y Sanidad Animal, Facultad de Veterinaria, Universidad Cardenal, Herrera-CEU, CEU Universities, C/Tirant lo Blanc, 7, Alfara del Patriarca, 46115 Valencia, Spain; 3 Department of Theriogenology, Faculty of Veterinary Medicine, Aswan University, Aswan 81528, Egypt; walaamohamed1995@yahoo.com; 4 Laboratory of Biotechnology, Department of Microbiology, Agriculture Research Center (ARC), Animal Health Research Institute (AHRI), Zagazig 44516, Egypt; dr.azzasalah@yahoo.com; 5 Department of Animal, Poultry, and Fish Production, Faculty of Agriculture, Damietta University, Damietta 34517, Egypt; ali21384@yahoo.com

**Keywords:** endometritis, probiotic, microbial metabolites, antibiotic, nanoencapsulation, buffalo, fertility

## Abstract

A *Lactococcus* (*L.*) *lactis* strain producing antimicrobial and anti-inflammatory biomolecules (mainly 1,4-Diaza-2,5-dioxobicyclo[4.3.0]nonanes and pyrazine-derivatives) was tested for its capacity to cure clinical endometritis in buffaloes compared to conventional antibiotic-based treatment. Clinical endometritis-diagnosed buffaloes (n = 16/group) were infused intrauterine with four doses of 10^9^ CFU-free (FLC group) or nanoencapsulated *L. lactis* (NLC group) and compared to those that received three doses of saline + a single dose of 500 mg cephapirin benzathin (AB group) or four doses of saline (control, C group) every other day. Endometrium samples were analyzed for cytological (polymorphonuclear cells, PMN), bacteriological, and proinflammatory mRNA expression. Uterine wash and blood samples were collected to determine proinflammatory cytokine concentrations and metabolites in the blood samples. The reproductive performance of buffaloes was assessed. Compared to the C group, the AB and NLC groups had the lowest percentage of PMN, followed by those in the FLC group (*p* < 0.05). All treated buffaloes had significantly lower numbers of pathogens than the control buffaloes. Compared to control, all treatments significantly down-regulated endometrial proinflammatory encoding mRNA expression. The concentrations of *IL1B*, *TNFAIP7*, and leukocyte esterase activity in the uterine washings were significantly decreased in the AB and NLC groups compared to the C and FLC groups. All treatments significantly decreased concentrations of serum proinflammatory cytokines compared to control. Both the AB and NLC groups had significantly lower concentrations of serum NEFA than the C and FLC groups. The percentage of control buffaloes having an echogenic uterus and PVD score > 2 was significantly higher than those in the treated buffaloes with higher numbers of corpora lutea, higher conception rates, and shorter days open than control buffaloes (*p* < 0.05). In conclusion, *L. lactis*-producing antimicrobial and anti-inflammatory metabolites reduce uterine inflammatory responses and improve fertility in buffaloes.

## 1. Introduction

In cattle, parturition and early postpartum periods are associated with increased risks of reproductive tract microbial dysbiosis, the emergence of inflammatory cascade interactions, and disruption of systemic immunity [1]. These events contribute to the weakening of the uterine defense mechanisms against invading pathogens, increasing the possibility of uterine diseases (endometritis, metritis, pyometra, and mucometra) [2]. Among uterine diseases, endometritis is a common reproductive disease that disrupts reproductive performance (for example, decreased first-service conception rates and increased embryonic losses) and causes significant economic losses on dairy farms [3]. This is because a healthy functional endometrium is required for the completion of several reproductive events, including luteolysis, maternal recognition of pregnancy, and early embryonic development and implantation [4]. In buffaloes, the reported clinical incidence of endometritis can be high (2.4%–20.68%), and clinical studies have recorded endometritis to be the most frequent reproductive disorder [5].

Conventionally, endometritis is treated by antibiotics (for example, penicillin, ampicillin, oxytetracycline, aminoglycosides, and sulfonamides) administered either systemically or intrauterine in a single dose or repeated doses [6]. Antibiotic-based therapies result in a wide range of animals’ responses, and in many cases, the antibiotic-based therapies are not specific against certain types of pathogens. Moreover, recently, there have been global concerns regarding the use of antibiotics for treating diseases in food-producing animals. These concerns involve the possibility of the transfer of antibiotic residues to humans through animal products and the emergence of antimicrobial resistance, threatening human and animal health [7,8].

Under this scenario, innovating antibiotic alternatives such as probiotic-based therapies seems like a promising therapy for tackling uterine diseases, including endometritis. Probiotics are a group of beneficial live microbes that can improve reproductive health via different mechanisms. It has been reported that probiotics can restore eubiosis in an infected uterus through different modes of action. Probiotics have antimicrobial, anti-inflammatory, and immunomodulatory effects [9]. Moreover, they can improve the reproductive tract environment and functioning through their ability to strengthen epithelial barrier functions of the reproductive tract, maintain vaginal acidity, and block the adherence of pathogens to uterine epthelial cells [10].

In the field of reproductive medicine, *Lactobacillus* species, *Bifidobacterium* species, and *Bacillus* species are the major probiotic candidates used for amending reproductive tract microbial dysbiosis [9]. Peter et al. [11] reported that intrauterine infusion of *Lactococcus* (*L.*) *buchneri* DSM 32407 (1.5–2 × 10^10^ CFU/animal) on days 24–30 postpartum improved the reproductive performance of cows with subclinical endometritis and healthy cows. Deng [12] reported that intravaginal infusion of a probiotic cocktail (composed of *L. sakei* FUA3089, *Pediococcus (P). acidilactici* FUA3138, and *P. acidilactici* FUA3140 with a cell count of 10^8^–10^9^ CFU/dose) reduced the incidence of uterine diseases (i.e., metritis, clinical endometritis, and pyometra) in dairy cows. Similarly, intravaginal infusion of a mixture of probiotics significantly reduced the incidence of endometrial inflammation in early postpartum dairy cows [13].

*Lactococcus lactis* subsp. *lactis* ATCC 11454 is one of the lactic acid bacteria strains isolated from anchu mash. This strain is identified by its capacity to produce nisin, a broad-spectrum bacteriocin that inhibits the growth of many bacteria, including *Bacillus* sp., *Clostridium* sp., *Brochothrix* sp., *Corynebacterium* sp., numerous *Lactobacillus* spp., *Enterobacter* sp., *Leuconostoc* sp., *Listeria* sp., *Micrococcus* sp., *Pediococcus* sp., *Staphylococcus* sp., and some Actinomyceae. Thus, this strain is included in dairy products to suppress the growth of pathogenic bacteria, mainly those of *Clostridium* sp. [14].

To the authors’ best knowledge, there are no available studies exploring the effects of probiotic-based therapies on the cure of clinical endometritis and subsequent reproductive performance in buffaloes. Therefore, this study examined the effects of intrauterine infusion of saline or a probiotic (*Lactococcus lactis*)-based therapy or a conventional antibiotic-based therapy on the inflammatory status and reproductive performance of buffaloes with clinical endometritis symptoms. Moreover, two forms of *L. lactis*, free or nanoencapsulated, were used to evaluate whether nanoencapsulation technology could improve the biological activity of *L. lactis* to cure endometritis-related symptoms and reproductive performance of infected buffaloes or not.

## 2. Materials and Methods

### 2.1. Preparation and Encapsulation of L. lactis

The preparation and encapsulation of *L. lactis* were carried out at the laboratory of nanoencapsulation and biotechnology, Animal and Fish Production Department, Faculty of Agriculture, Alexandria University, Egypt. The probiotic *L. lactis* ATCC 11454 was obtained from the Microbiological Resource Center, Faculty of Agriculture (MIRCEN, Ain Shams University, Cairo, Egypt). For mass production of *L. lactis*, De Man, Rogosa, and Sharpe (MRS, Merck, Darmstadt, Germany) agar and broth media were autoclaved at 120 °C and at 1–1.5 atm for 15 min. *L. lactis* was first activated by culturing on De MRS agar plates at 37 °C for 48 h. Then, the resultant colonies were inoculated in MRS broth under anaerobic conditions at 37 °C for 48 h. Wet biomass was collected by centrifugation at 5000 rpm for 15 min at 25 °C, washed three times with saline, re-suspended in a mixture of 85% MRS broth plus 15% glycerol, and kept at −80 °C. A sample of the resultant supernatant, cell-free supernatant, was extracted using 70% methanol for identifying metabolites synthesized by *L. lactis* using gas chromatography–mass spectroscopy (GC-MS) following the isolation conditions described by [15].

For *L. lactis* encapsulation, sodium alginate (Oxford La Fine Chem Lip, Maharashtra, India) and gum Arab (LOBA Chemie, Mumbai, India) were used to fabricate the coating wall, adopting the ionic-gelation method [16]. In brief, under continuous magnetic stirring, *L. lactis* (10^12^ CFU/mL) was first mixed with 50 mL of sodium alginate solution (1.5%, *w*/*v*). Then, the mixture was added dropwise using a syringe pump into a 100 mL Arab gum solution (3%, *w*/*v* at pH: 4.5). The encapsulated *L. lactis* was centrifuged at 8000 rpm for 20 min and collected. The survival rate of the postbiotic strain after freezing and thawing processes was determined and was considered when preparing doses of 10^9^ CFU each. The doses were stored at −80 °C for later application.

**Figure 1:**
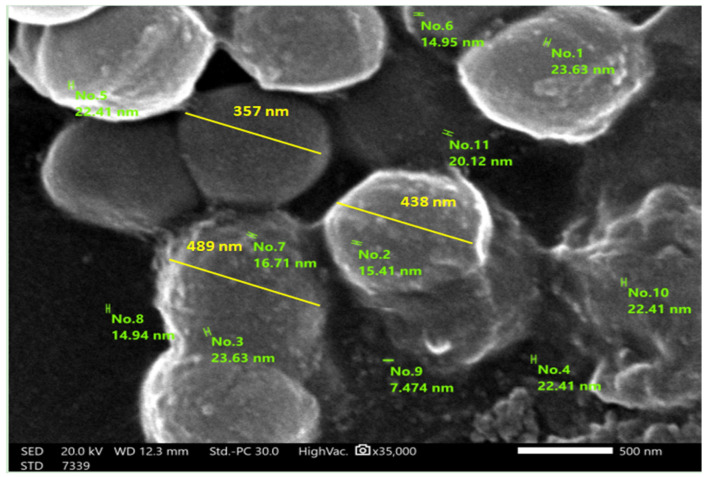
A scanning electron microscope (SEM) for alginate–gelatin nanoencapsulated *L. lactis* (yellow lines) showing the diameters of whole bacteria and sound wall material particles (green lines).

Scanning Electron Microscopy (SEM; Jeol JSM- 6360 LA, 3–1-2 Musashino, Akishima, Tokyo, Japan) was used to identify the morphology and size of the alginate–gum Arab nanoencapsulated *L. lactis*. The SEM image of alginate–gum Arab nanoencapsulated *L. lactis* showed that the average size of the wall material particles and whole nanoencapsulated bacteria were 18.55 ± 0.85 nm and 478.0 ± 10.85 nm, respectively (Figure 1).

### 2.2. Enrollment of Buffaloes and Treatment

The experimental protocol and all procedures applied to animals in this study were approved by Zagazig University—Institutional Animal Care and Use Committee (ZU-IACUC, approval number: ZU-IACUC/2/F/322/2023, the approval date: 27 September 2023). The study was conducted on the teaching and research farm of the Faculty of Veterinary Medicine, Aswan University. Buffaloes were maintained in a free-stall housing system and received their daily nutrient requirements [17]. Buffaloes had free access to tap water and salt blocks *ad libitum*. A total of 64 buffaloes, with an average age of 4–8 years, body weight of 350–450 kg, and body condition scores of 2.9 ± 0.75 (scale: 1 = thin and 5 = fat) [18], that possessed clinical endometritis were used. The diagnosis of clinical endometritis cases was based on the presence of purulent/muco-purulent vaginal discharge (PVD) on day 26 postpartum. The vaginal discharge of the buffaloes was determined with a Metricheck device (Metricheck, Simcro Tech, Hamilton, New Zealand) as defined by [19]. The vaginal discharge accumulated on the device was assessed visually with regards to volume, color, and proportion of pus. The character of PVD was assessed and classified as follows: 0 = no discharge, 1 = clear mucus, 2 = mucus with flecks of pus, 3 = mucopurulent discharge, 4 = purulent discharge, and 5 = foul-smelling discharge [3]. Clinical endometritis was declared in buffaloes with PVD score 2 through score 5. The PDV score means for the experimental groups were: 3.58, 3.78, 3.69, and 3.63 for the C group, AB group, FLC group, and NCL group, respectively (SEM = 0.271; *p* < 0.417). Buffaloes diagnosed with clinical endometritis were assigned to four experimental groups (n = 16/group). The first group (C group) served as a control where buffaloes received four intrauterine infusions of 50 mL of physiological saline (0.9% NaCl). The second group (AB group) included buffaloes treated with three doses of 50 mL of physiological saline and a single intrauterine infusion of 500 mg of cephapirin benzathin (Metricure, Intervet, Boxmeer, The Netherlands)/50 mL physiological saline. The third and fourth groups received four intrauterine infusions of 50 mL of physiological saline containing either 10^9^ CFU-free *L. lactis* or 10^9^ CFU-nanoencapsulated *L. lactis* (FLC and NLC groups) [12,13] (Ametaj et al., 2014 and Deng et al., 2016). The intrauterine infusion of saline, saline/antibiotic, and bacteria was applied every other day.

### 2.3. Endometrial Biopsy and Related Examinations

After one week of the end of the treatment protocols, samples from the endometrial epithelium were obtained with the cytobrush technique from the uterine body. Three cytobrush samples were collected. The first cytobrush was used for cytological analysis to determine the percentage of polymorphonuclear cells (PMN). The second cytobrush was used for bacteriological analysis [20]. The third cytobrush was placed in liquid nitrogen, transported to the laboratory, and stored at −80 °C until RNA isolation.

#### 2.3.1. Cytological Evaluation

Endometrial cytology was used to determine the percentage of endometrial PMN in the collected cytbruchs. Two smears of each sample were mounted on clean glass microscope slides and allowed to dry. Smears were fixed in absolute methanol for 10 min, stained with a modified Wright-Giemsa (Hema3, Biochemical Sciences, Swedesboro, NJ, USA) for 30 min, and microscopic evaluation was performed under a bright light microscope (B203, Soif Optical Instruments, Shanghai, China) at 400× magnification [21]. The percentage of PMN was determined in 2 different regions of each slide based on a differential count of 200 cells (PMN and endometrial cells).

#### 2.3.2. Microbiological Isolation

The cytobrush was transferred to a bijou bottle containing sterile phosphate buffer solution (PBS, pH 7) and pummeled for 2 min in a Stomacher (Seward Inc, Bohemia, NY, USA). Ten-fold serial dilutions in sterile buffer peptone were performed up to 10^7^, and aliquots of 0.1 mL of each dilution were pour-plated in Reinforced clostridial agar, Columbia blood agar, Edwards, Bile Esculin, Baird–Parker agar, Cetrimide Pseudomonas base agar, and Violet Red Bile Dextrose media (OXOID) to determine *Clostridia*, *Trueperella pyogenes*, *Streptococci*, *Bacteroides fragilis*, *Staphylococci*, *Pseudomonas aeruginosa*, and Coliforms, respectively. The plates were incubated at 37 ± 1 °C for 24 h under aerobic conditions for *Streptococci*, *Bacteroides fragilis*, *Staphylococci*, *Pseudomonas*, and *Coliforms* bacteria. For the anaerobic bacteria *Clostridium* spp. and *Trueperella pyogenes*, plates were incubated in anaerobic conditions at 37 °C for 3 days and at 35 °C for 5 days, respectively. Identification of bacteria was based on the characteristics of the colony, Gram stain, morphology, hemolysis, biochemical profile (API systems, BioMérieux, Basingstoke, UK), and other standard tests [22,23]. The results were expressed as the log number of colony-forming units per gram (Log CFU/g).

#### 2.3.3. Isolation of Total RNA and Reverse Transcription

Total RNA was isolated from cytobrush samples using the QIAamp RNeasy Mini kit (Qiagen, North Rhine-Westphalia, Germany) according to the manufacturer’s instructions. The concentration of the obtained RNA for each sample was measured using the compact Thermo Scientific NanoDrop Eight Spectrophotometer (Thermo Fisher Scientific, 168 Third Avenue, Waltham, MA, USA). β-actin was normalized as an internal control (housekeeping gene). Primers were supplied by Metabion, Germany. The sequence of each primer’s forward and reverse strands is shown in Table 1. Primers were utilized in a 20 µL reaction containing 10 µL of the 2× HERA SYBR^®^ Green RT-qPCR Master Mix (Willowfort, Birmingham, UK), 1 µL of RT Enzyme Mix (20×), 1 µL of each primer at a concentration of 20 pmol, 3 µL of water, and 5 µL of RNA template. The reaction was performed using a StepOne^TM^ real-time PCR machine in the Biotechnology Unit, Animal Health Research Institute, Zagazig Branch, Egypt. The cycling conditions consisted of an initial denaturation step of 94 °C for 15 min, followed by 40 cycles of 94 °C for 15 s, 55 °C for 30 s, and 72 °C for 30 s, with a final extension at 72 °C for 10 min. The PCR assay included a negative control containing the PCR mixture without an RNA template. The amplification curves and Ct values were determined by the StepOne software (StepOne™ and StepOnePlus™ Software v2.3, Thermo Fisher Scientific). To estimate the variation in gene expression from the RNA of the different samples, the cycle threshold (Ct) of each sample was compared with that of the positive control group using the “ΔΔCt” method using the ratio of 2^−ct^, where ΔCt = Ct [target gene] − Ct [housekeeping] [24,25].

### 2.4. Collection and Analysis of Uterine Lavage

After one week of the end of the treatment protocols, a volume of 50 mL of isotonic phosphate buffer solution was injected into the uterus, and washing solution was collected using a Foley catheter (Jorgen Kruuse, Marslev, Denmark) with a syringe. Transrectal massage of the uterus was performed before aspiration of at least 10 mL of the fluid, and the saline solution was aspirated by pulling back the syringe plunger to create negative pressure in the catheter. Uterine fluid was transferred to a sterile glass tube and centrifuged at 3000 rpm for 10 min at 4 °C, and the supernatant was transferred to 2-mL glass tubes and stored at −20 °C until analysis. Within 12 h of uterine washing collection, leukocyte esterase activity testing was performed by shaking the individual glass tube for 15 s and placing a drop of the solution on a leukocyte esterase commercial test strip (Siemens Lifestix^®^, Siemens Healthcare, Tokyo, Japan) with a pipette. The test result for every buffalo was read according to the manufacturer’s colorimetric chart after 2 min, and the score was recorded as follows: 0 = negative, 0.5 = trace of leukocytes, 1 = small amount of leukocytes, 2 = moderate amount of leukocytes, and 3 = large amount of leukocytes [30]. Uterine lavage samples were analyzed in duplicate samples for tumor necrosis factor-α (TNF-α), interleukin-β1 (IL-β1), and interleukin-6 (IL-6) concentrations using commercially available kits (Bovine Enzyme-Linked Imunosorbent Assay, ELISA, Sunred Biotechnology Company^®^, Shanghai, China) following the manufacturer’s guidelines. The assay ranges of bovine TNF-α, IL-β1, and IL-6 kits were between 15–4000 ng/L, 1.5–400 pg/mL, and 30–6000 ng/L, respectively. The sensitivities of bovine TNF-α, IL-β1, and IL-6 kits were 14.155 ng/L, 1.053 pg/mL, and 28.725 ng/L, respectively.

### 2.5. Blood Sampling

A blood sample (15 mL) was drawn from the jugular vein of each buffalo into a plain vacutainer tube seven days after the end of the treatment protocols. The samples were kept at +4 °C for two hours in the laboratory for clotting and then centrifuged at 3000 rpm for 15 min. The separated serum samples were transferred to the 1.5 mL glass tubes and stored at -20 °C until analysis. The concentrations of inflammatory cytokines, glucose, non-esterified fatty acids (NEFA), and insulin were determined. Concentrations of glucose (PGO enzyme preparation, Sigma Aldrich, Louis, MO, USA) and NEFA (NEFA-C^®^ kit; Wako Pure Chemical Industries, Osaka, Japan; intra- and inter-assay coefficients of variation were 2.0% and 2.9%, respectively) Insulin concentration was quantified as free insulin with a commercial ^125^I-IRMA kit validated for bovine samples (BI-Insulin IRMA kit; CIS Bio International, St Aubin, Ile De France, France). The sensitivity of the assay was 3.16 pmol/L; intra- and inter-assay CV was between 1.3% and 5.6% and ≤8.5%, respectively. Concentrations of TNF-α, IL-1β, and IL-6 in blood serum were measured in duplicate samples following the procedure mentioned previously.

### 2.6. Ultrasonographic and Vaginal Examination and Reproductive Performance

The PVD score was re-evaluated 7 days after the end of the treatment protocols, as described in the previous section. Buffaloes with PVD scores of less than 2 were considered positively responded cases (recovered from clinical endometritis), while those scored 2 or more were considered unresponsive cases (suffering from clinical endometritis symptoms). All buffaloes were subjected to ovarian and uterine transrectal ultrasonography scanning using a portable ultrasound with a 5.0/7.5 MHz linear transducer (Honda Electronincs, Toyohashi, Japan). Each uterine horn was assessed for fluid accumulation and the echogenicity of any uterine fluid. The uterine diameter was measured at approximately 10 cm cranial to the uterine bifurcation, while the diameter of the cervix was measured at the level of the external cervical os. The presence/absence of ovarian structures, mainly corpora lutea and dominant follicles, was recorded on day 9 of the estrous cycle for those animals that showed signs of estrus. A corpus luteum was defined as a grainy echogenic structure that had a well-defined border. A dominant follicle was defined as the largest follicle in the ovary with an internal diameter of ≥8 mm [31].

After the end of the voluntary waiting period (day 55–60 days pp), buffaloes were daily monitored for signs of overt estrus through visual observation by trained operators at 12 h intervals each day for 30 min at each observational period. Buffaloes that displayed any of the following signs of estrus: mounting other cows or standing to be mounted restlessly, licking/sniffing of the vulva, tail raising, frequent micturition, bellowing, or chin resting were considered to be in estrus and naturally inseminated with fertile bulls. The conception rate, expressed as a percentage, was estimated as the number of buffaloes diagnosed pregnant by rectal ultrasonography examination at day 35 post-mating divided by the number of buffaloes showing estrus and subjected to mating with fertile bulls. Days open were defined as the time interval between parturition and re-conception after first service (the calving-to-conception interval).

### 2.7. Statistical Analysis

The data were edited using Microsoft Excel (Microsoft Corporation, Redmond, WA, USA). To assess normality, the Levene and Shapiro–Wilk tests were conducted. The significant effects of the treatments were evaluated using general linear model analysis (PROC GLM; SAS Institute Inc., Cary, NC, USA; 2012), with the significance level (α) set at 0.05. If a significant effect was detected, pairwise comparisons between means were performed using Tukey’s test. Statistical significance between means was considered at a *p*-value below 0.05. The results were expressed as means ± standard error (SE). The binominal data related to uterine characteristics, ovarian activity, and reproductive performance were analyzed using the Chi-square test.

## 3. Results

### 3.1. Metabolites of L. lactis

The GC-MS analysis of the cell-free supernatant of *L. lactis* identified 25 metabolites. The major postbiotic metabolites were: 1,4-Diaza-2,5-dioxobicyclo[4.3.0]nonane; 3-isobutylhexahydropyrrolo[1,2-a]pyrazine-1,4-dione; 5,10-Diethoxy-2,3,7,8-tetrahydro-1h,6h-dipyrrolo[1,2-a:1,2-d]pyrazine; Hexadecanoic acid,2,3-dihydroxypropyl ester; and 2,7,12,17-Tetrakis(4-vinylphenyl)-, representing around 60% of total identified metabolites (Table 2).

### 3.2. Effects on Uterine Cytology and Microbiology

The effects of treatments on endometrium polymorphonuclear cells (PMN) and the numbers of pathogenic microbes in buffaloes with clinical endometritis are shown in Table 3. Compared to the C group, the AB and NLC groups showed the lowest significant (*p* < 0.05) percentages of PMN, followed by those in the FLC group. Buffaloes in the AB, FLC, and NLC had lower (*p* < 0.05) numbers of *Clostridia*, *Trueperella pyogenes*, *Streptococci*, *Bacteroides fragilis*, *Pseudomonas aeruginosa*, and *Coliforms* compared to buffaloes in the C group. There were no significant effects of different treatments on the numbers of *Staphylococci*.

### 3.3. Effects on Endometrium mRNA Expression

**Figure 2:**
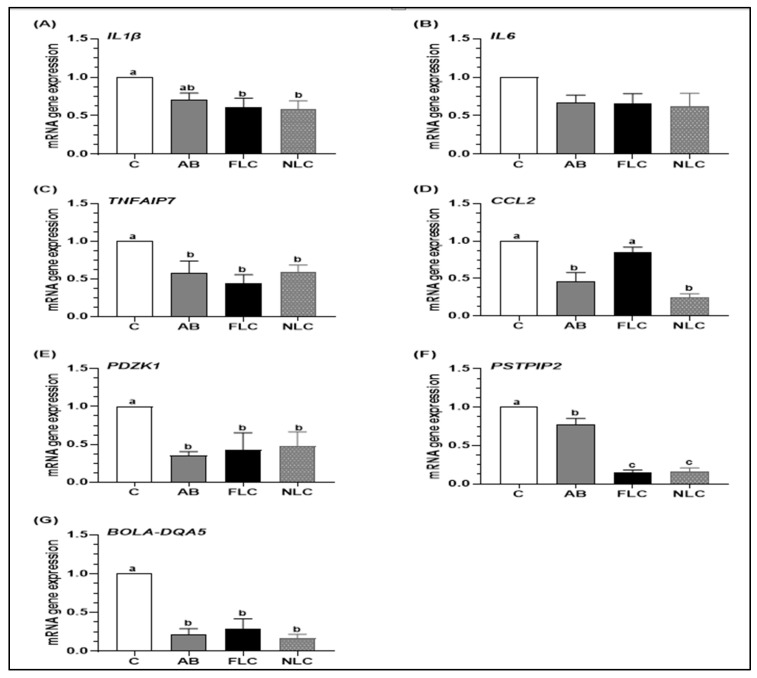
Effects of treatments (C: control, AB: 500 mg cephapirin benzathin, FLC: 10^9^ CFU-free *L. lactis*, and NLC: 10^9^ CFU nanoencapsulated *L. lactis*) on the mRNA expression level of (**A**) Interleukin-1β (*IL1β*), (**B**) Interleukin-6 (*IL6*), (**C**) Tumor necrosis factor alpha-induced protein 7 (*TNFAIP7*), (**D**) Chemokine (C-C motif) ligand 2 (*CCL2*), (**E**) PDZ Domain-containing 1 (*PDZK1*), (**F**) Proline-serine-threonine phosphatase-interacting protein 2 (*PSTPIP2*), and (**G**) Bovine leukocyte antigen-DQ alpha 5 (*BOLA-DQA5*). ^a,b,c^ Means with different superscript letters are significantly different at *p* < 0.05.

The effects of treatments on endometrium mRNA expression in buffaloes with clinical endometritis are presented in Figure 2. Both FLC and NLC groups had significantly lower mRNA expression for the *IL1β* gene than the C group, while the AB group showed an intermediate value (*p* < 0.05). Compared to the C group, all treatment groups significantly down-regulated the mRNA expression of *TNFAIP7*, PDZK, and *BOLA-DQA5* genes in the endometrium (*p* < 0.05). Both the AB and NLC groups had significantly lower (*p* < 0.05) mRNA expression for the *CCL2* gene than the C and FLC groups. Compared to the C group, the mRNA expression of the *PSTPIP2* gene in the endometrium was significantly downregulated in the FLC and NLC groups, followed by the AB group (*p* < 0.05).

### 3.4. Effects on Proinflammatory Cytokines in Uterine Washings

The effects of proinflammatory cytokine treatments and leukocyte esterase activity in the uterine washings of buffaloes with clinical endometritis are shown in Table 4. The concentrations of IL-1β in the uterine washings were significantly decreased in the AB and NLC groups compared to the C and FLC groups. The AB, FLC, and NLC groups had lower concentrations of IL-6 in the uterine washings than the C group, with the lowest value observed in the AB group, followed by those in the NLC and FLC groups (*p* < 0.05). The concentration of TNF-α in the uterine washings was significantly (*p* < 0.05) reduced in the AB and NLC groups compared to the C and FLC groups (*p* < 0.05). The leukocyte esterase activity was significantly reduced in the AB, FLC, and NLC groups compared to the C group, with the lowest values recorded in the AB and NLC groups.

### 3.5. Effect on Blood Serum Proinflammatory Cytokines and Energy Status-Related Metabolites

The effect of treatments on blood serum proinflammatory cytokines and energy status-related metabolites in buffaloes with clinical endometritis is shown in Table 5. Both the AB and NLC groups had significantly lower concentrations of IL-1β than the C group, whereas the differences in IL-1β concentrations were insignificant in the C and FLC groups. The AB, FLC, and NLC groups significantly (*p* < 0.05) decreased concentrations of blood serum proinflammatory cytokines (IL-6 and TNF-α) compared to the C group. Concentrations of these pro-inflammatory cytokines in the AB and NLC groups were similar. The AB, FLC, and NLC treatments did not affect (*p* < 0.05) the concentrations of blood serum glucose and insulin compared to the C group; however, both AB and NLC groups had significantly lower concentrations of blood serum NEFA than the C and FLC groups.

### 3.6. Effects on Uterine Characteristics, Ovarian Activity, and Reproductive Performance

The effects of treatments on uterine characteristics, ovarian activity, and reproductive performance of buffaloes with clinical endometritis are presented in Table 6. The percentage of buffaloes having echogenic uteri in the C group tended (*p* < 0.070) to be higher than those in the AB, FLC, and NLC groups. High percentages of buffaloes in the AB, FLC, and NLC groups had significantly lower uterine and cervical diameters than those in the C group. Similarly, buffaloes in the AB, FLC, and NLC groups had significantly lower numbers of dominant follicles and higher numbers of corpora lutea than control buffaloes (*p* < 0.05). Compared to control, buffaloes in the AB, FLC, and NLC groups (100% vs. 31.25%; *p* < 0.05) showed a PVD score <2, indicating a recovery from clinical endometritis symptoms. Estrous rate was not affected by the treatments (*p* > 0.05). Conception rates were significantly (*p* < 0.05) higher in all the AB, FLC, and NLC groups than in the C group. AB, FLC, and NLC treatments resulted in a significant (*p* < 0.05) reduction in days open compared to control, recording the shortest period in the AB and FLC groups.

## 4. Discussion

Endometritis is a reproductive disease that is mainly induced by reproductive tract microbial dysbiosis, activating inflammatory signaling cascades in the infected uterus. The incidence of endometritis is associated with the prevalence of specific species of pathogens, including *Escherichia coli*, *Trueperella pyogenes*, and *Fusobacterium necrophorum* [9]. Metagenomic analyses of the uterine microbiome in samples obtained with an endometrial cytobrush have revealed that clinical endometritis and metritis are associated with a lower microbial diversity, dominated by *Bacteroidetes*, *Fusobacterium*, and *Trueperella,* and a lower abundance of *Lactobacillus, Escherichia, Shigella, Schlegelella, Prevotella,* and Streptococcus [32,33]. In addition, the abundance of *Staphylococcus*, *Corynebacterium*, and *Anaerococcus* is associated with an increased risk of subclinical endometritis in postpartum cows [34]. This microbial dysbiosis up-regulates the mRNA expression of proinflammatory cytokine-encoding genes, such as those responsible for the biosynthesis of cluster of differentiation 14, toll-like receptor 4, interleukins, and *TNFA*, in the reproductive tissues and blood circulation, typically as observed in our study. Proinflammatory cytokines can trigger inflammation by promoting free radical damaging effects, cell adhesion, autoimmune responses, and migration of the inflammatory-producing cells to the inflamed area. All these events have negative effects on reproductive tissue integrity and functioning, disrupting postpartum fertility [35]. Similarly, in this study, buffaloes with clinical endometritis showed higher mRNA expression for proinflammatory cytokine-encoding genes, concentrations of proinflammatory cytokines, dominance of pathogens in uterine cytobrush samples, and lower postpartum fertility than antibiotic- or probiotic-treated buffaloes.

Conventionally, clinical or subclinical endometritis is tackled by systemic and/or intrauterine/intravaginal infusions of antibiotics. In this study, a single intrauterine infusion of 500 mg cephapirin benzathine to buffaloes with endometritis significantly lowered the proinflammatory cytokine concentrations, cured endometritis as indicated by the decreased percentages of PMN and PVD scores, and improved ovarian activity and conception rates compared to control buffaloes. Similarly, [36] found that 66.7% of buffaloes with subclinical endometritis treated with a single intrauterine infusion of cephapirin benzathine 40 days postpartum recovered, compared to 28.6% of untreated ones. In another study, [7] achieved a 100% cure rate and a 40–50% pregnancy rate when buffaloes with chorionic endometritis were intravenously injected with 2 gm of ciprofloxacin and 2.5 gm of metronidazole for five consecutive days. Despite the positive effects of antibiotic treatments, there are several concerns regarding the use of antibiotics in the reproductive medicine of food-producing animals. These include planned selection of antibiotic type based on antibiotic sensitivity tests, potential delivery of antibiotics to milk and milk withdrawal period, negative effects on fertility and reproductive tract microbial eubiosis, mixed responses, and increased production cost [8]. In addition, the emerging antimicrobial resistance and its hazardous consequences for human and animal health limit their use to control diseases, and thus new antibiotic alternatives must be developed [37].

In this study, we aimed to cure endometritis by restoring postpartum reproductive tract microbial eubiosis using probiotics, eliminating the use of antibiotics. In fact, the use of probiotics to restore reproductive tract health and functioning is initially based on the positive relationships observed between the abundance of some microbial species normally existing in the reproductive tract of healthy cattle, mainly *Lactobacillus*, and reproductive tract functioning and fertility [9,38]. *Lactobacillus* species produce bioactive molecules such as hydrogen peroxide and lactic acid, which have been found to inhibit the growth of *Staphylococcus aureus* and *Trueperella pyogenes* that are commonly isolated from cattle suffering from uterine diseases [39]. Moreover, it has been found that a mixture of *Lactobacillus bacteria*, *Lactobacillus sakei*, and *Pediococcus acidilactici*, infused intravaginally as probiotics, significantly reduced the incidence of endometrial inflammation in early postpartum dairy cows [13].

In this study, we evaluated the potential of the probiotic *L. lactis* from the *Lactococcus* genus of lactic acid bacteria as a substitute for antibiotic-based treatment for curing clinical endometritis in buffaloes. This type of probiotic is known for its ability to produce different bacteriocins and bacteriocin-like inhibitory substances. These substances have bacteriostatic and bactericidal effects against pathogenic strains isolated from an animal origin (pig samples) such as *Escherichia coli*, *Salmonella enterica*, *Streptococcus suis*, *Streptococcus dysgalactiae*, *Staphylococcus hyicus*, and *Enterococcus faecalis* [40]. In this context, *L. lactis* has been reported as an effective therapy to treat mastitis in dairy cows, substituting antibiotic therapies [41]. In this study, for the first time, GC-MS analysis was used to identify the metabolites synthesized by *L. lactis* and to understand the mechanisms by which *L. lactis* can restore microbial eubiosis and the health of the uterus. According to the analysis, the major identified compound in *L. lactis* metabolites was 1,4-Diaza-2,5-dioxobicyclo[4.3.0]nonane. It has been found that such a compound possesses strong antimicrobial activity against *Staphylococcus aureus*, *Escherichia coli*, and antioxidant activity [42]. Moreover, this compound has the ability to prevent the formation of bacterial biofilm needed for the completion of pathogen adhesion and infection activities [43]. Various nitrogen-based heterocycles, such as triazoles, pyrazoles, pyrroles, imidazoles, pyridine, pyrazine, and pyrimidine, have been reported in the scientific databases as possessing multiple physiological and pharmacological properties. *L. lactis* used in this study produces numerous of these compounds. For example, the second and third abundant metabolites were pyrazine derivatives, which have been classified as non-peptidic antimicrobial and antioxidant compounds [44]. Moreover, a porphyrin derivative, 5,10,15,20-Tetrakis-(4-vinyl-phenyl)-porphyrin, with antibacterial, antibrotozoal, and anticancer activities, was identified [45,46,47]. Similarly, a triazole derived compound, 1, 4, 7-Triazaheptane, 1,7-bis(1-methyl-1-phosphonato)ethyl-, has been detected. Triazole derivatives have a broad range of biological activities, including antibacterial, antifungal, antitumor, anti-inflammatory, antitubercular, hypoglycemic, antidepressant, anticonvulsant, analgesic, antiviral, anticancer, antimalarial, and antioxidant [48]. Additionally, several short- and long-chain fatty acid derivatives with anti-inflammatory and antimicrobial activities were detected. These results might explain the reduced microbial load of pathogenic bacteria, including *Clostridia*, *Trueperella pyogenes*, *Streptococci*, *Bacteroides fragilis*, *Staphylococci*, *Pseudomonas aeruginosa*, and *Coliforms*, in the probiotic-treated buffaloes, which is comparable to the effects of the antibiotic cephapirin benzathin. Moreover, probiotic-treated buffaloes had lower concentrations of proinflammatory cytokines in uterine washings and blood serum, which were associated with lower mRNA expression of the proinflammatory cytokine-encoding genes in uterine tissues. These findings support the biological activity of different metabolites synthesized by *L. lactis* to relieve endometritis symptoms via different modes of action.

Introducing a new microbial strain to a non-commensal environment might hamper its colonization and competitive ability with other existing pathogens. Thus, we tested whether the nanoencapsulation process gave the probiotic more ability to survive and colonize the infected uteri. Specifically, inflammatory reproductive diseases can shift reproductive tract pH into alkalinity, altering the ideal environment of lactic acid bacteria, which prefer an acidic environment [49]. Nagy et al. [50] reported that the encapsulation of probiotics tailored for vaginal disease treatment using polyvinylalcohol nanofibers was an effective strategy to maintain the viability of vaginally administered probiotic-based products for a longer time in the reproductive tract with acceptable survival. Results of our study showed that NLC treatment, nanoencapsulated *L. lactis*, significantly reduced the concentrations of several proinflammatory cytokines such as IL-6 and TNF-α, leukocyte esterase activity in uterine washings, and NEFA in blood serum compared to FLC. Furthermore, these effects were comparable or sometimes stronger than the antibiotic effects. These findings may support the importance of the encapsulation process in improving the colonization ability of *L. lactis* and its survival for a long time when introduced to infected uteri with unpreferable harboring conditions. Additionally, the nanoencapsulation process may be necessary at the industrial/farm scales by allowing the applicant to store the probiotic-based products for a long time with an acceptable survival rate and biological activity.

In this study, significant reductions in NEFA concentrations in the AB- and NLC-treated buffaloes were observed. In dairy cows, intravaginal infusion of lactic acid bacteria cocktail (composed of *L. sakei* FUA3089, *P. acidilactici* FUA3138, and *P. acidilactici* FUA3140 with a cell count of 10^8^–10^9^ CFU/dose) lowered concentrations of NEFA in serum and the incidence of metritis and total uterine infections (i.e., metritis, clinical endometritis, and pyometra) [12]. In fact, the exact modes of action behind the reductions in NEFA concentrations observed in this study in the AB and NLC groups are not completely known. However, it might be attributed to the significant decrease in proinflammatory cytokines in these groups compared to those observed in the FLC and C groups. It was observed that injections of recombinant bovine TNF, a proinflammatory cytokine, can induce remarkable changes in the concentrations of blood plasma glucose, triglycerides, and NEFA in Holstein heifers [51]. Generally, the reduction in this metabolite is associated with better immune functions, energy status, and reproductive performance in dairy animals [52]. For example, prolonged exposure to circulating NEFA and β-hydroxybutyrate during late prepartum and early postpartum periods was associated with impairment of PMN function during the transition period in dairy cows [53]. Moreover, it was noticed that cows with NEFA ≥0.3 mm one week prepartum had nearly twice the odds of developing metritis [54]. Similarly, it has been reported that cows with NEFA >0.6 mm two weeks postpartum had a higher risk of metritis [55].

Dairy animals with uterine inflammatory diseases often undergo several reproductive disorders, including delayed uterine involution, oocyte incompetence, embryonic losses, and disrupted folliculogenesis and steroidogenesis [1]. Cows with longer durations of uterine infections possess decreased conception and pregnancy rates and extended days open [56]. In this study, buffaloes treated either with antibiotics or *L. lactis* in free or nanoencapsulated forms expressed higher numbers of corpora lutea and fewer large follicles than control buffaloes, indicating the occurrence of ovulation. They also had lower uterine and cervical diameters, percentages of PMN, and PVD scores, indicating the incidence of uterine involution and recovery. These improvements in ovarian and uterine functions were associated with higher conception rates and fewer days open compared to untreated buffaloes. This can be mainly attributed to the reduction in the pathogenic bacterial load and proinflammatory cytokine concentrations, leading to improved uterine health and functioning [57].

## 5. Conclusions

Endometritis in buffaloes is mainly caused by reproductive tract microbial dysbiosis and weakened immune function during the postpartum period. In this study, antibiotic-based treatment of a single dose of 500 mg cephapirin benzathin and probiotic-based treatments of 10^9^ CFU-free or nanoencapsulated *L. lactis* can restore reproductive microbial eubiosis and modulate the release of proinflammatory cytokines. Considering the hazards of antibiotic use in food-producing animals, preference could be directed to probiotic-based therapies using *L. coccus.* As indicated by GC-MS analysis, this microbial strain produces a wide range of metabolites with antimicrobial, antioxidant, anti-inflammatory, and anti-biofilm formation activities; thus, it presents an effective and safe solution for the treatment of clinical endometritis in buffaloes. However, future studies have to explore the metabolites secreted by the introduced probiotic strain in the uterine environment for a better understanding of the modes of action that might mediate their effects. Moreover, as seen in this study, the application of nanoencapsulation technology empowers the biological activity of *L. coccus*. Further studies are required to identify microbial species that can be used for treating reproductive diseases, particularly at the field scale, focusing on their metabolites and related biological pathways.

## Figures and Tables

**Table 1:** Target genes and primer sequences used for RT-PCR analyses.

Target Gene Name	Primer Sequences (5′–3′)	References
Beta-actin (*ACTB*)	F: CGTGGGCCGCCCTAGGCACCA	[26]
	R: GGGGGCCTCGGTCAGCAGCAC	
Interleukin-1β (*IL1B*)	F: GCCTTCAATAACTGTGGAACCAAT	[27]
	R:GTATATTTCAGGCTTGGTGAAAGGA	
Interleukin-6 (*IL6*)	F: GGCTCCCATGATTGTGGTAGTT	
	R: GCCCAGTGGACAGGTTTCTG	
Tumor Necrosis Factor alpha-induced protein 7 (*TNFAIP7*)	F: CGGTGGTGGGACTCGTATG	
	R: CTGGTTGTCTTCCAGCTTCACA	
Chemokine (C-C motif) ligand 2 (*CCL2*)	F: CCTCCTGTGCCTGCTAC	[28]
	R: TTGCTGCTGGTGACTCTT	
PDZ Domain-containing 1 (*PDZK1*)	F: AGCCCACAGTACAGCCTCTC	[29]
	R: CTCTGCAGTAGCCACACCTG	
Proline-serine-threonine phosphatase-interacting protein 2 (*PSTPIP2*)	F: ATAAGGTGCTGCTGGAAGA	
	R: CTGAAATCCCTGAGGACCTG	
Bovine leukocyte antigen-DQ alpha 5 (*BOLA-DQA5*)	F: CAGATGCACTGCCCATCTAT	
	R: CAGGGAGAGAATTCTGAGGG	

**Table 2:** Cell-free metabolites, postbiotic, of *L. lactis* detected by gas chromatography–mass spectrometry (GC–MS).

Compound Name	Area, %
1,4-Diaza-2,5-dioxobicyclo[4.3.0]nonane	29.26
3-Isobutylhexahydropyrrolo[1,2-a]pyrazine-1,4-dione	13.61
5,10-Diethoxy-2,3,7,8-tetrahydro-1h,6h-dipyrrolo[1,2-a:1,2-d]pyrazine	6.58
Hexadecanoic acid,2,3-dihydroxypropyl ester	5.78
5,10,15,20-Tetrakis-(4-vinyl-phenyl)-porphyrin	5.1
1, 4, 7-Triazaheptane, 1,7-bis(1-methyl-1-phosphonato)ethyl-	4.56
1-Dodecanol,3,7,11-trimethyl-	3.46
4-Octadecenal (spectrumdisagrees)	3.42
2H-Pyran,tetrahydro-2-(12-pentadecynyloxy)-	3.17
Dimethyldiphenyltethylidylpyrrolidine	2.96
Pregn-4-ene-3,20-dione,17,21-dihydroxy-,bis(o-methyloxime)	2.17
3-(6-Amino-4-oxo-1,4-dihydro-pyrimidin-2-ylsulfanyl)-propionic acid	2.05
2-Phenylmalonic acid	1.75
S-(1,3-Diphenylbutyl)dimethylthiocarbamate	1.74
Methyl9,10-dideutero-9-octadecenoate	1.61
Octadec-9-enoic acid	1.56
Octadecanoic acid,2,3-dihydroxypropyl ester	1.53
12,15-Octadecadiynoicacid, methyl ester	1.44
Phenol,2,4-bis(1,1-dimethylethyl)-	1.12
12,15-Octadecadiynoicacid, methyl ester	0.98
2-(5-[1,3]Dioxolan-2-yl-pentyl)-3-methyl-aziridine	0.88
À-d-Galactopyranoside,methyl2,3-bis-o-(trimethylsilyl)-,cyclic phenylboronate	0.8
Ethanimidothioic acid,2-(dimethylamino)-n-[[(methylamino)carbonyl]oxy]-2-oxo-, methyl ester	0.73
7-Oxooctanoic acid	0.73
1,1,3,3,5,5,7,7,9,9,11,11-Dodecamethyl-hexasiloxane	0.59
Benzeneethanamine,n,à,à-trimethyl-	0.58
Methylperdeuterio-hexadeca-7,10,13-trienoate	0.52
1,1,3,3,5,5,7,7,9,9,11,11,13,13,15,15-Hexadecamethyloctasiloxane	0.48

**Table 3:** Effects of treatments (C: control, AB: 500 mg cephapirin benzathin, FLC: 1 × 10^9^ CFU-free *L. lactis*, and NLC: 1 × 10^9^ CFU nanoencapsulated *L. lactis*) on endometrium polymorphonuclear cells (PMNS) and micro-pathological profile of buffaloes with clinical endometritis.

Items ^1^	Experimental Groups (n = 16/group)				SEM	*p*-Value
	C	AB	FLC	NLC		
PMNS, %	7.40 ^a^	3.40 ^c^	5.00 ^b^	4.00 ^c^	0.342	<0.001
Microbiological test, Log CFU/g						
*Clostridia*	6.47 ^a^	4.01 ^b^	3.06 ^b^	3.13 ^b^	0.547	0.007
*Trueperella pyogenes*	5.74 ^a^	1.56 ^b^	1.40 ^b^	1.04 ^b^	0.228	0.002
*Streptococci*	6.14 ^a^	2.68 ^b^	3.45 ^b^	1.81 ^b^	0.352	0.006
*Bacteroides fragilis*	3.50 ^a^	2.08 ^b^	1.76 ^b^	1.70 ^b^	0.181	<0.001
*Staphylococci*	3.88	2.83	3.08	2.26	0.706	0.479
*Pseudomonas aeruginosa*	2.76 ^a^	1.84 ^b^	1.11 ^c^	1.44 ^bc^	0.061	0.012
*Coliforms*	5.29 ^a^	2.72 ^b^	1.47 ^b^	1.23 ^b^	0.389	0.005

^a,b,c^ Means with different superscript letters are significantly different at *p* < 0.05. ^1^ PMNS = polymorphonuclear cells.

**Table 4:** Effects of treatments (C: control, AB: 500 mg cephapirin benzathin, FLC: 10^9^ CFU-free *L. lactis*, and NLC: 10^9^ CFU nanoencapsulated *L. lactis*) on uterine wash proinflammatory cytokines and leukocyte esterase activity of buffaloes with clinical endometritis.

Items ^1^	Experimental Groups (n = 16/group)				SEM	*p*-Value
	C	AB	FLC	NLC		
IL-1β, pg/mL	27.11 ^a^	13.28 ^b^	22.29 ^a^	14.00 ^b^	2.465	0.003
IL-6, pg/mL	215.31 ^a^	105.84 ^d^	191.34 ^b^	128.99 ^c^	7.598	<0.001
TNF-α, pg/mL	126.78 ^a^	69.96 ^b^	111.91 ^a^	81.29 ^b^	5.570	<0.001
Leukocyte esterase	3.80 ^a^	1.20 ^c^	2.20 ^b^	1.40 ^c^	0.308	<0.001

^1^ IL-1β = Interleukin-1β, IL-6 = Interleukin-6, and TNF-α = Tumor necrosis factor alpha. ^a,b,c,d^ Means with different superscript letters are significantly different at *p* < 0.05.

**Table 5:** Effects of treatments (C: control, AB: 500 mg cephapirin benzathin, FLC: 10^9^ CFU-free *L. lactis*, and NLC: 10^9^ CFU nanoencapsulated *L. lactis*) on serum proinflammatory cytokines and energy status-related metabolites of buffaloes with clinical endometritis.

Items ^1^	Experimental Groups (n = 16/group)				SEM	*p*-Value
	C	AB	FLC	NLC		
Proinflammatory cytokines						
IL-1β, pg/mL	31.78 ^a^	14.38 ^c^	28.58 ^ab^	19.09 ^bc^	3.869	0.020
IL-6, pg/mL	228.49 ^a^	151.86 ^b^	227.99 ^a^	174.01 ^b^	9.864	<0.001
TNF-α, pg/mL	218.76 ^a^	90.32 ^c^	165.06 ^b^	102.70 ^c^	9.086	<0.001
Energy-related metabolites						
Glucose, mg/dL	84.03	86.13	80.39	79.89	3.737	0.345
Insulin, µU/mL	8.634	11.13	8.81	9.23	1.058	0.351
NEFA, mg/dL	5.04 ^a^	2.99 ^b^	5.60 ^a^	3.24 ^b^	0.388	<0.001

^1^ IL-1β = Interleukin-1β, IL-6 = Interleukin-6, TNF-α = Tumor necrosis factor alpha, and NEFA = non-esterified fatty acids. ^a,b,c^ Means with different superscript letters are significantly different at *p* < 0.05.

**Table 6:** Effects of treatments (C: control, AB: 500 mg cephapirin benzathin, FLC: 10^9^ CFU-free *L. lactis*, and NLC: 10^9^ CFU nanoencapsulated *L. lactis*) on uterine characteristics, ovarian activity, and reproductive performance of buffaloes with clinical endometritis.

Items ^1^	Experimental Groups				*p*-Value
	C	AB	FLC	NLC	
Intrauterine fluid, %					
Absent	56.25 (9/16)	81.25 (13/16)	87.50 (14/16)	93.75 (15/16)	0.070
An echogenic	43.75 (7/16)	18.75 (3/16)	12.50 (2/16)	6.25 (1/16)	
Uterine horn diameter, cm					
<3.5	12.50 ^b^ (2/16)	62.50 ^a^ (10/16)	50.00 ^ab^ (8/16)	37.50 ^ab^ (6/16)	0.045
3.5–5	37.50 (6/16)	25.00 (4/16)	31.25 (5/16)	37.50 (6/16)	
>5	50.00 (8/16)	12.50 (2/16)	18.75 (3/16)	25.00 (4/16)	
Cervical diameter, cm					
<5	12.50 (2/16)	25.00 (4/16)	31.25 (5/16)	18.75 (3/16)	0.027
5–7	12.50 ^b^ (2/16)	56.25 ^a^ (9/16)	43.75 ^ab^ (7/16)	50.00 ^ab^ (8/16)	
>7	75.00 ^a^ (12/16)	18.75 ^b^ (3/16)	25.00 ^b^ (4/16)	31.25 ^b^ (5/16)	
Ovarian function					
No. of ovulatory follicles, >8 mm	0.81 ^a^	0.50 ^b^	0.44 ^b^	0.56 ^b^	
No. of corpora lutea	0.25 ^b^	0.44 ^a^	0.50 ^a^	0.56 ^a^	
PVD score (%)					<0.001
<2	31.25 ^b^ (5/16)	100.00 ^a^ (16/16)	100.00 ^a^ (16/16)	100.00 ^a^ (16/16)	
>2	68.75 ^a^ (11/16)	00.00 ^b^ (0/16)	00.00 ^b^ (0/16)	00.00 ^b^ (0/16)	
Reproductive performance					
Estrous rate,%	75.00 (12/16)	81.25 (13/16)	62.50 (10/16)	68.75 (11/16)	0.672
Conception rate, %	16.66 ^b^ (2/12)	69.23 ^a^ (9/13)	70.00 ^a^ (7/10)	72.72 ^a^ (8/11)	0.015
Days open	150 ^a^	80 ^c^	95 ^c^	105 ^bc^	<0.001

^1^ PVD: 0 = no discharge, 1 = clear mucus, 2 = mucus with flecks of pus, 3 = mucopurulent discharge, 4 = purulent discharge, or 5 = foul-smelling discharge. ^a,b,c^ Means with different superscript letters are significantly different at *p* < 0.05.

## Data Availability

The raw data supporting the conclusions of this article will be made available by the authors on request.
